# Pediatric lung transplantation in China, 2019–2023

**DOI:** 10.1007/s12519-025-00916-4

**Published:** 2025-06-03

**Authors:** Xiao-Shan Li, Zi-Tao Wang, Bo Wu, Shu-Gao Ye, Feng Liu, Chun-Xiao Hu, Yi Lu, Wen-Jie Hua, Wei-Wei Xu, Man Huang, Jing-Yu Chen

**Affiliations:** 1 Department of Lung Transplantation Center, The Affiliated Wuxi People’s Hospital of Nanjing Medical University, Wuxi People’s Hospital, Wuxi Medical Center, Nanjing Medical University, Wuxi, 214023, China; 2 Chinese Lung Transplantation Quality Management and Control Center, Wuxi, 214023, China; 3 Department of Transplant Anesthesiology, The Affiliated Wuxi People’s Hospital of Nanjing Medical University, Wuxi People’s Hospital, Wuxi Medical Center, Nanjing Medical University, Wuxi, 214023 China; 4 Wuxi School of Medicine, Jiangnan University, Wuxi, 214122, China; 5 Department of General Intensive Care Unit, The Second Affiliated Hospital of Zhejiang University School of Medicine, Hangzhou, 310052, China; 6 Key Laboratory of Early Warning and Intervention of Multiple Organ Failure, Ministry of Education of the People’s Republic of China, Hangzhou, 310052, China

**Keywords:** Indication, Lung transplantation in China, Pediatric lung transplantation, Survival

## Abstract

**Background:**

Pediatric lung transplant (pLTX) is a rare procedure globally; its characteristics and survival outcomes in China remain unknown.

**Methods:**

This retrospective study analyzed data from pLTX recipients aged ≤ 17 years between January 2019 and December 2023 from the China Lung Transplantation Registry. Pre-, intra-, and post-operative characteristics were described and compared between children aged 2–11 years and 12–17 years and between pLTX conducted in centers with high and low transplant volumes. The Kaplan‒Meier method was used to estimate the postoperative survival rates and 95% confidence intervals (CIs). One-year postoperative survival rates were compared between pediatric and adult lung transplant (LTX) patients via log-rank tests.

**Results:**

Between 2019 and 2023, 63 transplants were performed in 62 pediatric patients, accounting for 1.8% of the total LTX in China. The primary indication for pLTX was bronchiolitis obliterans syndrome (46.0%), followed by cystic fibrosis (12.7%) and idiopathic pulmonary arterial hypertension (11.1%). Infection was the most common complication after pLTX (63.9%), and the incidence of bronchial anastomotic stenosis was slightly higher among recipients aged 2–11 years than among those aged 12–17 years (14.3% vs. 2.9%, *P* = 0.244). High-volume hospitals had a higher incidence of infections (72.7% vs. 41.2%, *P* = 0.021) and primary graft failure (20.0% vs. 5.9%, *P* = 0.260) among pediatric recipients. However, acute rejection was exclusively observed in low-volume hospitals (0.0% vs. 17.6%, *P* = 0.018). The in-hospital mortality rate was 16.1% (95% CI = 6.7–25.5). The 30-day and one-year survival rates after pLTX were 93.5% (95% CI = 87.6–99.9) and 80.6% (95% CI = 71.4–91.1), respectively, and were significantly higher than those of adult recipients (82.0% and 58.7%, all *P* < 0.05).

**Conclusions:**

This research identified the trends, indications, donor and recipient characteristics, and complications of pLTX in China. Despite its small size, pLTX is growing gradually and has favorable outcomes. Future research on the long-term follow-up of pLTX recipients is needed to identify factors associated with the prognosis of pLTX patients.

**Graphical Abstract:**

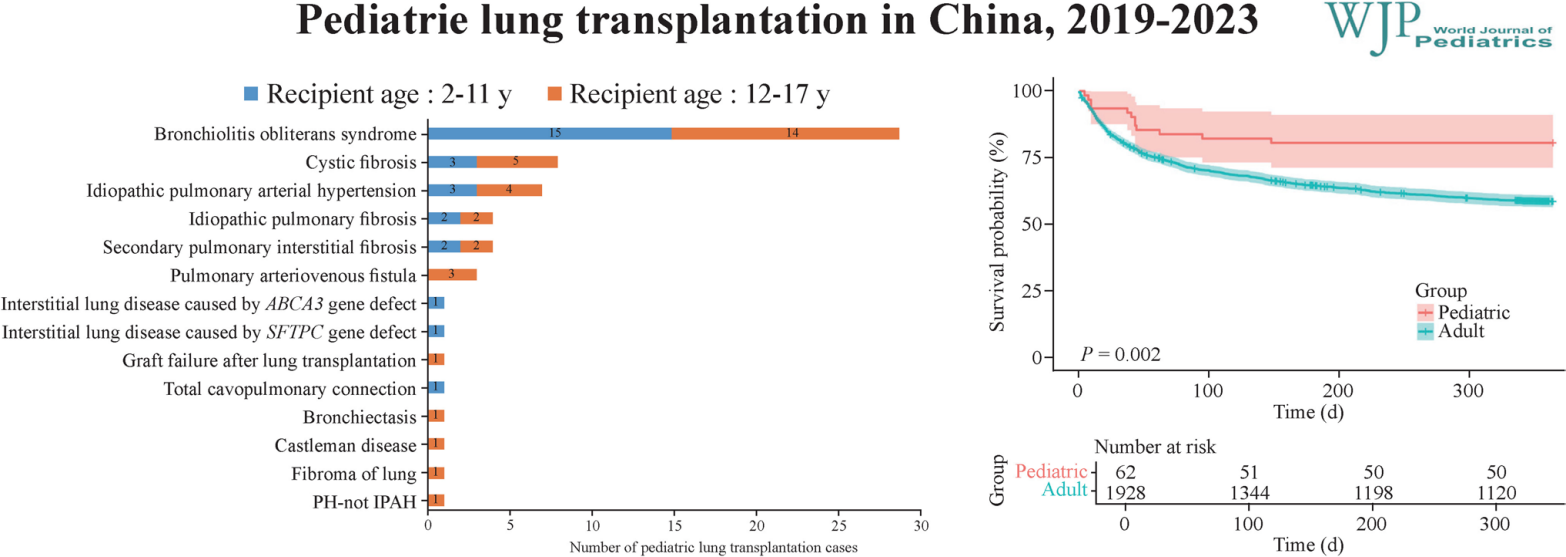

**Supplementary Information:**

The online version contains supplementary material available at 10.1007/s12519-025-00916-4.

## Introduction

Lung transplantation (LTX) represents a therapeutic option for patients suffering from a variety of end-stage lung diseases, offering the potential for prolonging life expectancy and improving quality of life. According to the International Society for Heart and Lung Transplantation (ISHLT), the total number of adult lung transplantations (aLTX) worldwide has experienced a threefold increase over time, from nearly 11,000 in the 1990s to approximately 34,000 between 2010 and 2018 [1]. In China, 1053 adult patients received LTX between 2015 and 2018 [2], and the annual number of LTX cases is still growing due to the rapid development of organ preservation methods and surgical techniques as well as the constant refinement of immunosuppressive regimens [2].

In contrast to aLTX, pediatric lung transplantation (pLTX) remains a relatively rare procedure, with fewer than three thousand transplantations performed among patients under 18 years of age reported to the ISHLT Pediatric Thoracic Transplant Registry in the past three decades [3]. The development of pLTX is hindered by several critical factors, including limited access to organ donors, challenges in achieving donor-recipient matching compatibility, difficulties in postoperative management, and poor survival prognosis.

Yue et al*.* previously reported ten cases of pediatric patients who underwent LTX at a single LTX center in China [4]. In recent years, there has been a growing number of pLTX in China, but the recipient profiles, operation characteristics, and outcomes after pLTX have not been fully investigated.

In this study, we described the characteristics and outcomes of pLTX in 62 patients across 13 transplant centers in China between 2019 and 2023, with the aim of providing insights into the latest developments and experience in the field of pLTX in China.

## Methods

### Study design

Sixty-two pediatric recipients aged ≤ 17 years who underwent LTX at 13 hospitals in China between January 2019 and December 2023 were identified and included in this retrospective cohort study. This study was approved by the Medical Ethics Committee of the Affiliated Wuxi People’s Hospital of Nanjing Medical University (approval number: KY25014). Written informed consent was waived because this was a retrospective secondary analysis of deidentified data. All procedures performed in this research involving human participants were in accordance with the Declaration of Helsinki (as revised in 2013).

### Data source

The characteristics of the donors and recipients and the survival of the pediatric recipients following LTX were obtained from the China Lung Transplantation Registry (CLuTR, https://clutr.cotr.cn/login.jsp). CLuTR represents a unique registry system for LTX in China that comprehensively collects data on pre-operative, intra-operative, post-operative, and follow-up information of recipients and basic information of donors in a timely manner. CLuTR has been committed to analyzing LTX-related data in a dynamic and scientific manner, setting the scientific foundation for national regulatory authorities to develop transplantation-related policies and regulations. We thoroughly reviewed the data obtained from the CLuTR and cross-verified the accuracy of the information with the corresponding reporting hospitals. To compare post-transplant survival rates between pediatric and adult patients, we additionally retrieved information on aLTX recipients during the same study period (January 2019-December 2023). Because almost all pediatric patients underwent bilateral LTX in this study (96.8%), we included only survival data from adults with bilateral LTX in the analysis. The outcomes of retransplantation or a history of other solid organ transplantation in children or adult patients were not included in the survival analysis.

All grafts came from voluntary donation after the citizen’s death, and the next of kin of the donor provided written informed consent with free will. The Institutional Ethics Committees of the Organ Procurement Organization (OPO) approved the donation procedures. Donor lungs were allocated through the China Organ Transplant Response System, with comprehensive consideration of the recipient body size, blood type, urgency status, and the time already spent on the waiting list (https://www.cot.org.cn/).

In China, LTX can be performed only by hospitals with qualifications approved by the National Health Commission according to the Regulations on the Registration and Administration of Human Organ Transplantation Clinical Practices [5]. To date, a total of 60 hospitals nationwide are authorized to perform LTX in China. Because no hospitals or surgeons specialize solely in pLTX, all pLTX surgeries are performed at institutions with extensive experience in aLTX. Despite China’s multi-tiered medical system, many patients seek medical care in higher-tier hospitals without a referral, particularly for complex procedures such as LTX.

### Indications, procedures, and postoperative care

Pediatric patients being considered for LTX are required to meet the criteria set for conventional LTX. Because the specific criteria for recipients and donors in pLTX have not been established in China, the guidelines for aLTX were therefore applied, which have been previously described [2]. Each case was reviewed and approved by the Institutional Lung Transplant Evaluation Committee. Donor retrieval was performed following the standard procedure [6, 7]. Celsior and Perfadex were used as perfusates accordingly, and donor lungs were preserved and transported on ice until transplantation.

The use of extracorporeal membrane oxygenation (ECMO) in pLTX followed both expert consensus on the use of ECMO in pediatric critical care in China [8, 9] and international guidelines from the ISHLT [10]. Briefly, for children who require extracorporeal life support during surgery, ECMO is favored rather than cardiopulmonary bypass, and different modes [veno-venous (V-V), veno-arterial (V-A), or veno-arteriovenous] are applied accordingly. Pneumonectomy was performed after the establishment of ECMO. The transplant procedure used for pLTX was largely the same as that used for adults. Briefly, anastomosis was performed following the sequence of the bronchus, pulmonary artery, and left atrium sleeve. The peripheral tissue around the bronchus was reconstructed for the prevention of anastomotic fistula. Lobar transplant or donor size reduction was optional. Intra- and post-operative immunosuppressive therapy included methylprednisolone and tacrolimus.

### Outcomes

The outcomes included 30-day, 90-day, 180-day, and one-year postoperative survival rates; in-hospital mortality; acute rejection within 30 days after transplantation; and complications following LTX. In the current study, all deaths were due to LTX-related complications.

### Follow-up

All patients were followed up at 30 days, 180 days, and one year after LTX and then annually after the first year. All patients were followed up from the date of LTX surgery to death or at least one year after LTX.

### Statistical analysis

All the statistical analyses and plots were performed in R software version 4.0.2. A two-sided test was adopted, and *P* < 0.05 was considered significant. Continuous variables with a normal distribution are described by the mean ± standard deviation (SD), and those that deviated from a normal distribution or were severely skewed are described by the median and interquartile range (IQR). Categorical variables and ordinal variables are presented as counts (*n*) and percentages (%). The differences in indications between age groups (2–11 and 12–17 years) were compared via Fisher’s exact tests via Monte Carlo estimation. Patients were divided into two age groups according to donor lung allocation rules in China: 2–11 years and 12–17 years. Hospitals that reached more than 10 cases of pLTX from 2019–2023 were categorized as high-volume centers, whereas the remaining hospitals were classified as low-volume centers. The postoperative characteristics of the pLTX were compared between these two groups. The Kaplan‒Meier method was used to estimate the postoperative survival rates and 95% confidence intervals (CIs) and to plot the survival curves for each group of recipients. We compared one-year postoperative survival rates between pLTX and aLTX patients via log-rank tests. Differences in in-hospital mortality and one-year mortality by characteristics of pediatric recipients were analyzed via negative binomial regression models and univariate Cox regression, respectively.

## Results

### Trends in pediatric lung transplantation in China

**Fig. 1:**
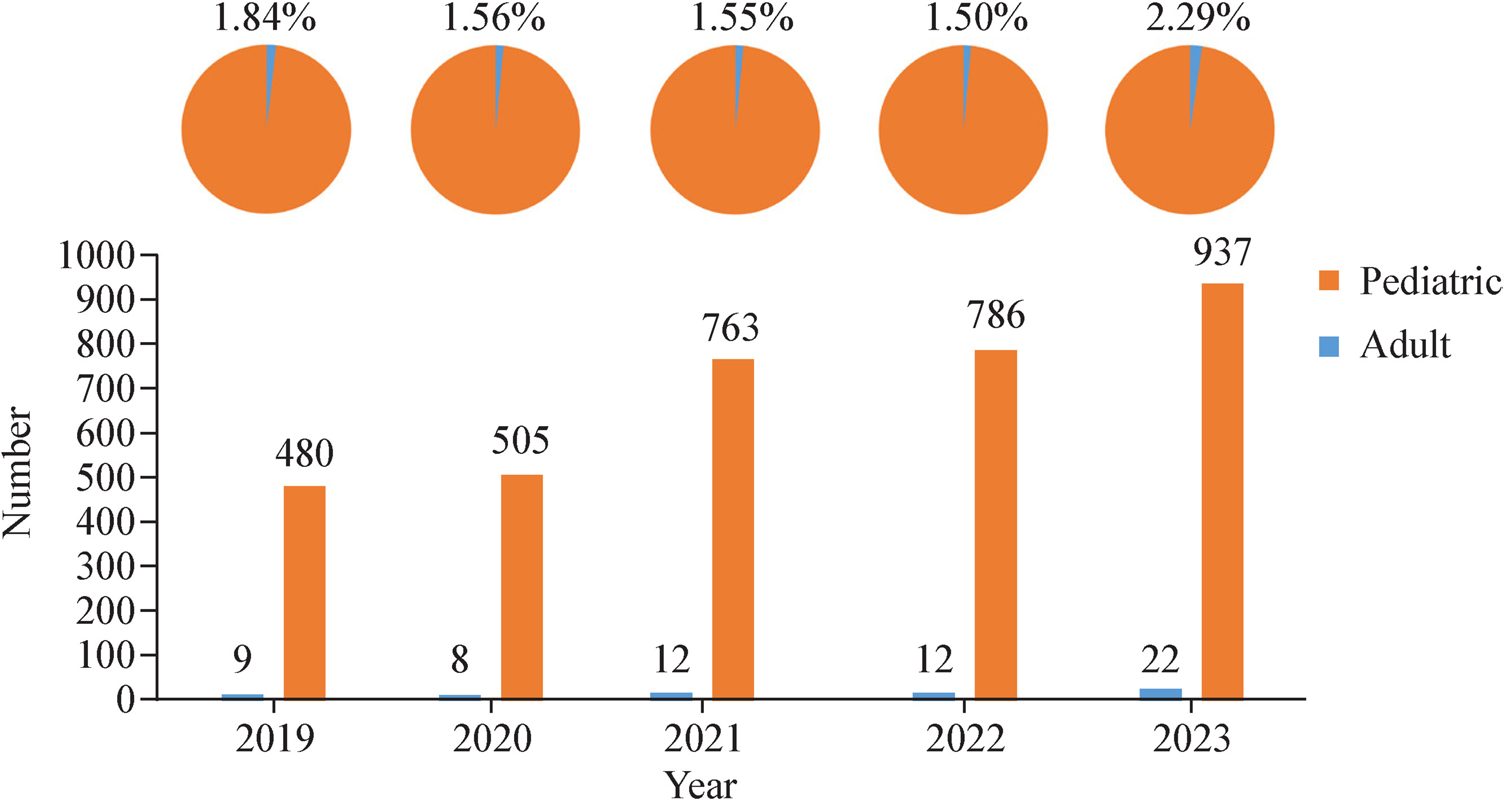
Number of pediatric and adult lung transplants in China from 2019–2023

Between January 2019 and December 2023, 63 pLTX procedures were performed in 62 pediatric patients in China. As shown in Fig. 1, the number of pLTX in each year was 9, 8, 12, 12, and 22 from 2019 to 2023, accounting for 1.84%, 1.56%, 1.55%, 1.50%, and 2.29%, respectively, of the total LTX in each corresponding year.

pLTX was performed in 13 hospitals in China between 2019 and 2023. The majority of transplants (73.0%) were performed in centers with over 100 cases of LTX annually (Supplementary Fig. 1). Over eighty percent of pLTX procedures for recipients between 2 and 11 years of age (82.1%, 23/28) were performed at Wuxi People’s Hospital and the Second Affiliated Hospital of Zhejiang University School of Medicine. The distribution of recipient age was not significantly different across hospitals (*P* = 0.716).

**Fig. 2:**
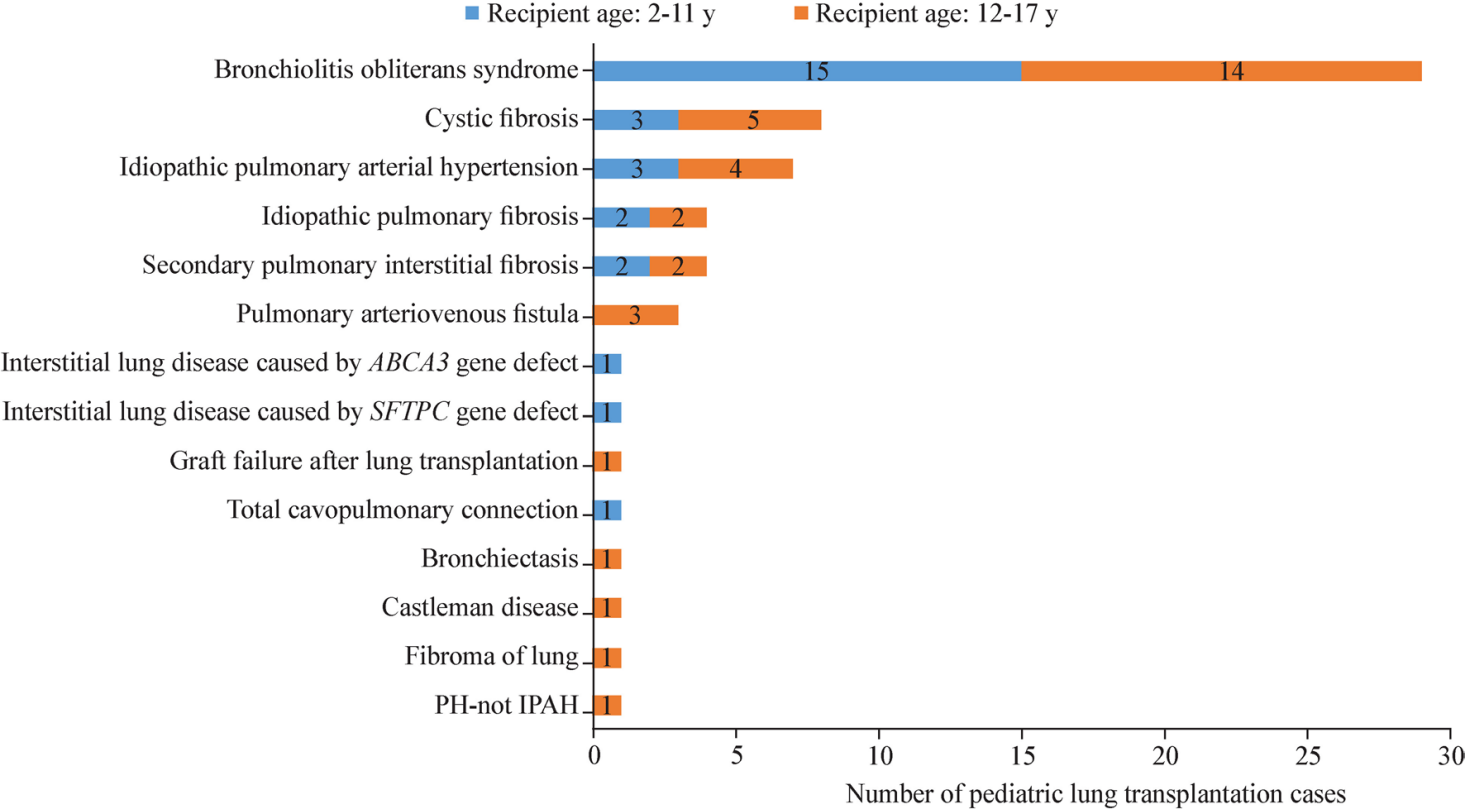
Distribution of indications for pediatric lung transplantation cases in China from January 2019 to December 2023. *PH* pulmonary hypertension *IPAH* idiopathic pulmonary arterial hypertension

Among the 63 pLTX cases, bronchiolitis obliterans syndrome (BOS) was the primary indication, accounting for 46.0%, followed by cystic fibrosis (CF, 12.7%), idiopathic pulmonary arterial hypertension (IPAH, 11.1%), idiopathic pulmonary fibrosis (6.3%), secondary pulmonary interstitial fibrosis (6.3%), and pulmonary arteriovenous fistula (4.8%) (Fig. 2). The indications did not differ significantly between recipients aged 2–11 years and those aged 12–17 years (*P* = 0.757).

### Characteristics of pediatric lung transplantation recipients

The demographics and preoperative clinical characteristics of pLTX recipients overall and by recipient age group in China between 2019 and 2023 are presented in Table 1. Among the 62 recipients, 48.4% were males, and females accounted for 51.6%. The body mass index (BMI) among recipients aged 2–11 years (13.8 ± 3.4 kg/m^2^) was lower than that among those aged 12–17 years (16.2 ± 3.7 kg/m^2^, *P* = 0.013). The proportion of types of hospitalization was significantly different between the two groups. The median (IQR) level of creatinine among recipients aged 2–11 years was 24.1 (19.9–35.0) µmol/L, which was significantly lower than that among those aged 12–17 years [median (IQR): 41.0 (32.4–54.2), *P* < 0.001]. We did not observe significant differences in other characteristics by age group (Table 1).

**Table 1:** Preoperative characteristics of pediatric lung transplant recipients overall and by recipient age groups in China, 2019–2023

Characteristics	Total (*N* = 62)	Recipient age (y)		Statistics	*P*
		2–11 (*n* = 28)	12–17 (*n* = 34)		
Male, *n* (%)	30 (48.4)	13 (46.4)	17 (50.0)	0.078	0.779
BMI (kg/m^2^), mean ± SD	15.1 ± 3.7	13.8 ± 3.4	16.2 ± 3.7	2.561	0.013
Blood type, *n* (%)					
A	17 (27.4)	9 (32.1)	8 (23.5)	–	0.544
B	13 (21.0)	5 (17.9)	8 (23.5)		
AB	8 (12.9)	5 (17.9)	3 (8.8)		
O	24 (38.7)	9 (32.1)	15 (44.1)		
Hospitalization, *n* (%)					
Not hospitalized	6 (9.7)	2 (7.1)	4 (11.8)	2.095	0.036
General hospitalization	44 (71.0)	17 (60.7)	27 (79.4)		
ICU	12 (19.4)	9 (32.1)	3 (8.8)		
Grade of NYHA heart function, *n* (%)					
NYHA III	42 (67.7)	19 (67.9)	23 (67.6)	0.293	0.769
NYHA IV	4 (6.5)	0 (0.0)	4 (11.8)		
Severe and requires hospitalization	16 (25.8)	9 (32.1)	7 (20.6)		
Oxygen inhalation, *n* (%)	55 (88.7)	23 (82.1)	32 (94.1)	–	0.228
Non-invasive ventilation, *n* (%)	8 (12.9)	4 (14.3)	4 (11.8)	–	1.000
Invasive mechanical ventilation, *n* (%)	11 (17.7)	6 (21.4)	5 (14.7)	–	0.523
ECMO usage, *n* (%)	4 (6.5)	1 (3.6)	3 (8.8)	–	0.620
Recent systolic blood pressure (mmHg), mean ± SD	109.9 ± 15.2	106.9 ± 14.4	112.3 ± 15.6	1.401	0.166
Recent diastolic blood pressure (mmHg), mean ± SD	66.7 ± 10.6	66.3 ± 10.8	67.0 ± 10.6	0.272	0.786
Ejection fraction (%), mean ± SD	65.1 ± 7.7	65.3 ± 7.1	64.9 ± 8.2	0.172	0.864
Right atrial enlargement, *n* (%)	11 (18.0)	5 (17.9)	6 (18.2)	0.001	0.974
Right ventricular enlargement, *n* (%)	12 (19.7)	5 (17.9)	7 (21.2)	0.108	0.743
Creatinine (µmol/L), median (IQR)	34.5 (22.2–47.2)	24.1 (19.9–35.0)	41.0 (32.4–54.2)	3.700	< 0.001
Total bilirubin (µmol/L), median (IQR)	8.8 (5.9–10.9)	8.5 (5.9–10.1)	9.3 (6.0–15.6)	0.962	0.336
Glutamic-pyruvic transaminase (U/L), median (IQR)	17.6 (12.0–31.6)	17.5 (12.0–26.2)	17.6 (10.2–36.2)	0.071	0.944
Glutamic-oxaloacetic transaminase (U/L), median (IQR)	26.0 (19.0–39.5)	29.0 (24.0–38.5)	24.5 (17.2–41.0)	1.416	0.157
PaO_2_ (mmHg), median (IQR)	71.7 (52.9–104.4)	66.5 (53.2–92.5)	75.7 (53.3–114.2)	0.776	0.438
PaCO_2_ (mmHg), median (IQR)	49.5 (37.2–67.1)	51.8 (44.8–71.5)	44.5 (36.8–58.2)	1.850	0.064
FiO_2_ (%), median (IQR)	35.0 (29.0–45.0)	37.0 (29.5–45.0)	33.0 (29.0–41.0)	0.747	0.455
Oxygenation index (mmHg), median (IQR)	242.9 (160.6–281.2)	231.6 (143.7–281.7)	247.6 (196.0–278.1)	1.176	0.240
Pulmonary sepsis requiring treatment 2 or more times in the last year, *n* (%)	5 (8.1)	2 (7.1)	3 (8.8)	–	1.000
Steroid dependence (≥ 5 mg/d), *n* (%)	20 (32.3)	10 (35.7)	10 (29.4)	0.279	0.597
Pan-drug-resistant bacteria infection, *n* (%)	8 (12.9)	2 (7.1)	6 (17.6)	–	0.276
Anticoagulant usage, *n* (%)	4 (6.5)	1 (3.6)	3 (8.8)	0.101	0.750
Cordarone usage, *n* (%)	1 (1.6)	0 (0.0)	1 (2.9)	–	1.000
Pneumothorax, *n* (%)	3 (4.8)	1 (3.6)	2 (5.9)	–	1.000
Thoracotomy, *n* (%)	2 (3.2)	0 (0.0)	2 (5.9)	–	0.497
Diabetes mellitus, *n* (%)	2 (3.2)	0 (0.0)	2 (5.9)	–	0.497
Dialysis, *n* (%)	1 (1.6)	0 (0.0)	1 (2.9)	–	1.000
Gastroesophageal reflux, *n* (%)	1 (1.6)	0 (0.0)	1 (2.9)	–	1.000
History of chest surgery, *n* (%)	5 (8.1)	1 (3.6)	4 (11.8)	–	0.366
Hypertension, *n* (%)	2 (3.2)	1 (3.6)	1 (2.9)	–	1.000
Cerebrovascular disease, *n* (%)	1 (1.6)	0 (0.0)	1 (2.9)	–	1.000
Peripheral vascular disease, *n* (%)	1 (1.6)	0 (0.0)	1 (2.9)	–	1.000
Pulmonary embolism, *n* (%)	3 (4.8)	0 (0.0)	3 (8.8)	–	0.245
History of blood transfusion, *n* (%)	15 (24.2)	8 (28.6)	7 (20.6)	0.534	0.465
Tumor history (excluding skin cutaneous melanoma), *n* (%)	8 (12.9)	4 (14.3)	4 (11.8)	–	1.000
CMV-IgG+ , *n* (%)	11 (19.0)	5 (19.2)	6 (18.8)	–	1.000
CMV-IgM+ , *n* (%)	2 (3.5)	0 (0.0)	2 (6.5)	–	0.495
HBsAg+ , *n* (%)	1 (1.6)	1 (3.6)	0 (0.0)	–	0.452
HBcAb+ , *n* (%)	9 (14.5)	4 (14.3)	5 (14.7)	–	1.000
*Acinetobacter baumanii* infection, *n* (%)	6 (9.7)	2 (7.1)	4 (11.8)	–	0.681
*Pseudomonas aeruginosa* infection, *n* (%)	7 (11.3)	2 (7.1)	5 (14.7)	–	0.442
*Escherichia coli* infection, *n* (%)	3 (4.8)	1 (3.6)	2 (5.9)	–	1.000
Methicillin resistant *Staphylococcus aureus* infection, *n* (%)	2 (3.2)	0 (0.0)	2 (5.9)	–	0.497
Filamentous fungus infection, *n* (%)	3 (4.8)	0 (0.0)	3 (8.8)	–	0.245
*Candida albicans* infection, *n* (%)	1 (1.6)	0 (0.0)	1 (2.9)	1.000	0.246

*BMI* body mass index, *ICU* intensive care unit, *NYHA* New York Heart Association, *ECMO* extracorporeal membrane oxygenation, *PaO*_*2*_ partial pressure of arterial oxygen, *PaCO*_*2*_ partial pressure of arterial carbon dioxide, *FiO*_*2*_ fraction of inspired oxygen, *CMV* cytomegalovirus, *IgG* immunoglobulin G, *IgM* immunoglobulin M, *HBsAg* hepatitis B surface antigen, *HBcAb* hepatitis B core antibody, *SD* standard deviation, *IQR* interquartile range

### Characteristics of pediatric lung transplantation donors

Table 2 presents the demographics and clinical characteristics of pLTX donors overall and by recipient age group. One recipient received a lung retransplantation, and data on the donor of the second LTX were not included in the current analysis. Among the 62 donors, the median (IQR) age was 15.0 (8.2–30.5) years, and the age ranged between 1 and 51 years. Adult donor lungs from two pLTX recipients aged 2–11 years and 21 pLTX recipients aged 12–17 years were used. The age (*P* < 0.001) and BMI (*P* = 0.001) of the donors were significantly greater among older recipients (12–17 years) than among younger recipients (2–11 years). We did not observe significant differences in other donor characteristics by recipient age group (Table 2).

**Table 2:** Characteristics of pediatric lung transplant donors overall and by recipient age groups in China, 2019–2023

Characteristics	Total (*N* = 62)	Recipient age (y)		Statistics	*P*
		2–11 (*n* = 28)	12–17 (*n* = 34)		
Age (y), median (IQR)	15.0 (8.2–30.5)	8.0 (6.0–11.2)	26.5 (15.0–45.0)	5.296	< 0.001
Age (y), range	1–51	1–43	1–51		
Male, *n* (%)	43 (69.4)	17 (60.7)	26 (76.5)	1.794	0.180
BMI (kg/m^2^), mean ± SD	20.0 ± 5.3	17.7 ± 5.7	21.9 ± 4.1	3.332	0.001
Blood type, *n* (%)					
A	13 (21.0)	8 (28.6)	5 (14.7)	–	0.450
B	13 (21.0)	6 (21.4)	7 (20.6)		
AB	2 (3.2)	0 (0.0)	2 (5.9)		
O	34 (54.8)	14 (50.0)	20 (58.8)		
Donor type, *n* (%)					
Donation after brain death	60 (96.8)	27 (96.4)	33 (97.1)	–	1.000
Donation after cardio-brain death	2 (3.2)	1 (3.6)	1 (2.9)		
Oxygenation index (mmHg), mean ± SD	437.9 ± 78.1	442.1 ± 84.8	434.5 ± 73.3	0.376	0.709
Duration of ventilator usage (d), median (IQR)	6.5 (3.0–11.0)	6.0 (3.0–11.5)	7.0 (4.0–10.0)	0.268	0.789
Cold ischemia time (h), mean ± SD	7.3 ± 2.3	7.2 ± 2.2	7.3 ± 2.4	0.102	0.919

*BMI* body mass index, *IQR* interquartile range, *SD* standard deviation

### Intraoperative characteristics of pediatric lung transplantation recipients

Table 3 summarizes the intraoperative characteristics of pLTX recipients in China between 2019 and 2023. The total operation time was 5.7 ± 1.6 hours on average and ranged from 3.0 to 12.0 hours. A longer mean operation time was observed among recipients aged 12–17 years (6.1 ± 1.8 hours) than among those aged 2–11 years (5.2 ± 1.2 hours, *P* = 0.025). Compared with younger recipients, older recipients had a greater volume of blood loss (*P* = 0.001), volume of other fluid transfusions (*P* < 0.001), and urine volume (*P* = 0.001) (Table 3).

**Table 3:** Intraoperative characteristics of pediatric lung transplant recipients overall and by recipient age groups in China, 2019–2023

Characteristics	Total (*N* = 62)	Recipient age (y)		Statistic	*P*
		2–11 (*n* = 28)	12–17 (*n* = 34)		
Total operation time (h), mean ± SD	5.7 ± 1.6	5.2 ± 1.2	6.1 ± 1.8	2.292	0.025
Emergency lung transplantation, *n* (%)	10 (16.1)	5 (17.9)	5 (14.7)	–	0.744
Bilateral transplant, *n* (%)	60 (96.8)	28 (100.0)	32 (94.1)	–	0.497
ECMO usage, *n* (%)	43 (69.4)	17 (60.7)	26 (76.5)	1.794	0.180
Duration of ECMO usage (h), median (IQR)	5.8 (4.9–7.0)	5.5 (5.0–7.5)	5.9 (4.8–7.0)	0.324	0.746
Type of ECMO, *n* (%)					
V-A/V-A-V	24 (55.8)	10 (58.8)	14 (53.8)	0.103	0.748
V-V	19 (44.2)	7 (41.2)	12 (46.2)		
Volume of blood loss (L), median (IQR)	5.0 (3.0–10.0)	3.9 (2.0–6.0)	8.0 (4.2–11.4)	3.350	0.001
Volume of blood transfusion (L), median (IQR)	7.0 (3.8–13.6)	6.0 (3.5–10.6)	9.6 (4.5–20.9)	1.842	0.066
Volume of other fluid transfusion (L), median (IQR)	13.5 (9.1–25.0)	10..0 (6.8–11.8)	21.3 (13.6–31.1)	3.953	< 0.001
Urine volume (L), median (IQR)	9.0 (4.5–12.7)	6.0 (3.0–9.3)	12.0 (7.3–15.0)	3.243	0.001
Cardiac arrest, *n* (%)	2 (3.2)	1 (3.6)	1 (2.9)	–	1.000
Arrhythmias, *n* (%)	7 (11.3)	5 (17.9)	2 (5.9)	–	0.228
Hypotension, *n* (%)	8 (12.9)	3 (10.7)	5 (14.7)	–	0.719
Hematorrhea, *n* (%)	2 (3.2)	0 (0.0)	2 (5.9)	–	0.497
Pulmonary artery stenosis/embolism, *n* (%)	1 (1.6)	0 (0.0)	1 (2.9)	–	1.000
Hypoxemia, *n* (%)	3 (4.8)	1 (3.6)	2 (5.9)	–	1.000

*ECMO* extracorporeal membrane oxygenation, *V-A* venoarterial, *V-A-V* veno-venoarterial, *V-V* veno-venous, *IQR* interquartile range, *SD* standard deviation

### Preoperative, intraoperative, and postoperative ECMO usage

Four recipients required preoperative ECMO support, which was maintained throughout and after surgery. All recipients, including one below 12 years of age, were supported with V-V ECMO (Table 1). Intraoperatively, 43 (69.4%) recipients required ECMO support, with an overall median (IQR) duration of ECMO use of 5.8 (4.9–7.0) hours. There was no significant difference in intraoperative ECMO usage (60.7% vs. 76.5%, *P* = 0.180) or the median (IQR) duration of ECMO support [5.5 (5.0–7.5) hours vs. 5.9 (4.8–7.0) hours, *P* = 0.746] between those aged 2–11 years and those aged 12–17 years (Table 3). ECMO was used among 29 recipients after surgery (46.8%), with a median (IQR) duration of ECMO usage of 32.0 (16.0–133.5) hours. The use of ECMO was more common among recipients aged 12–17 years than among those aged 2–11 years (61.8% vs. 28.6%, *P* = 0.009). However, there was no significant difference in the median postoperative ECMO duration between the two groups [24.0 (16.0–77.2) hours vs. 96.0 (33.2–196.0) hours, *P* = 0.259] (Table 4).

**Table 4:** Postoperative characteristics of pediatric lung transplant recipients overall and by recipient age groups in China, 2019–2023

Characteristics	Total (*N* = 62)	Recipient age (y)		Statistic	*P*
		2–11 (*n* = 28)	12–17 (*n* = 34)		
Discharged alive, *n* (%)	52 (83.9)	22 (78.6)	30 (88.2)	–	0.326
Hospitalization duration (d), median (IQR)	33.5 (21.0–54.0)	26.5 (18.5–35.0)	38.5 (25.0–56.0)	1.974	0.048
ICU length of day (d), median (IQR)	6.7 (3.7–13.9)	6.4 (3.7–13.8)	7.0 (3.8–14)	0.057	0.954
Time between surgery and resumption of diet (h), median (IQR)	41.0 (24.0–72.0)	46.0 (38.0–72.0)	35.5 (22.8–72.5)	0.883	0.377
ECMO usage after surgery, *n* (%)	29 (46.8)	8 (28.6)	21 (61.8)	6.795	0.009
Duration of ECMO usage after surgery (h), median (IQR)	32.0 (16.0–133.5)	96.0 (33.2–196.0)	24.0 (16.0–77.2)	1.129	0.259
Reintubation, *n* (%)^a^	10 (16.4)	4 (14.8)	6 (17.6)	–	1.000
Duration of reintubation (d), median (IQR)	10.5 (3.7–26.0)	19.4 (6.3–37.4)	10.5 (2.2–18.3)	19.000	0.412

*ICU* intensive care unit, *ECMO* extracorporeal membrane oxygenation, *IQR* interquartile range. ^a^One heart–lung transplant was excluded

### Postoperative characteristics of pediatric lung transplantation recipients

Table 4 presents the postoperative characteristics of pLTX recipients overall and by age group from 2019–2023. Among 62 recipients, 52 survived to hospital discharge, and the survival rate at hospital discharge was 83.9%. The median (IQR) length of hospital stay and intensive care unit (ICU) stay among 52 survivors was 33.5 (21.0–54.0) days and 6.7 (3.7–13.9) days, respectively, and the mean (IQR) time between surgery and the resumption of diet was 41.0 (24.0–74.0) hours. Reintubation was performed in 10 recipients, with a median (IQR) duration of reintubation of 10.5 (3.7–26.0) days.

**Fig. 3:**
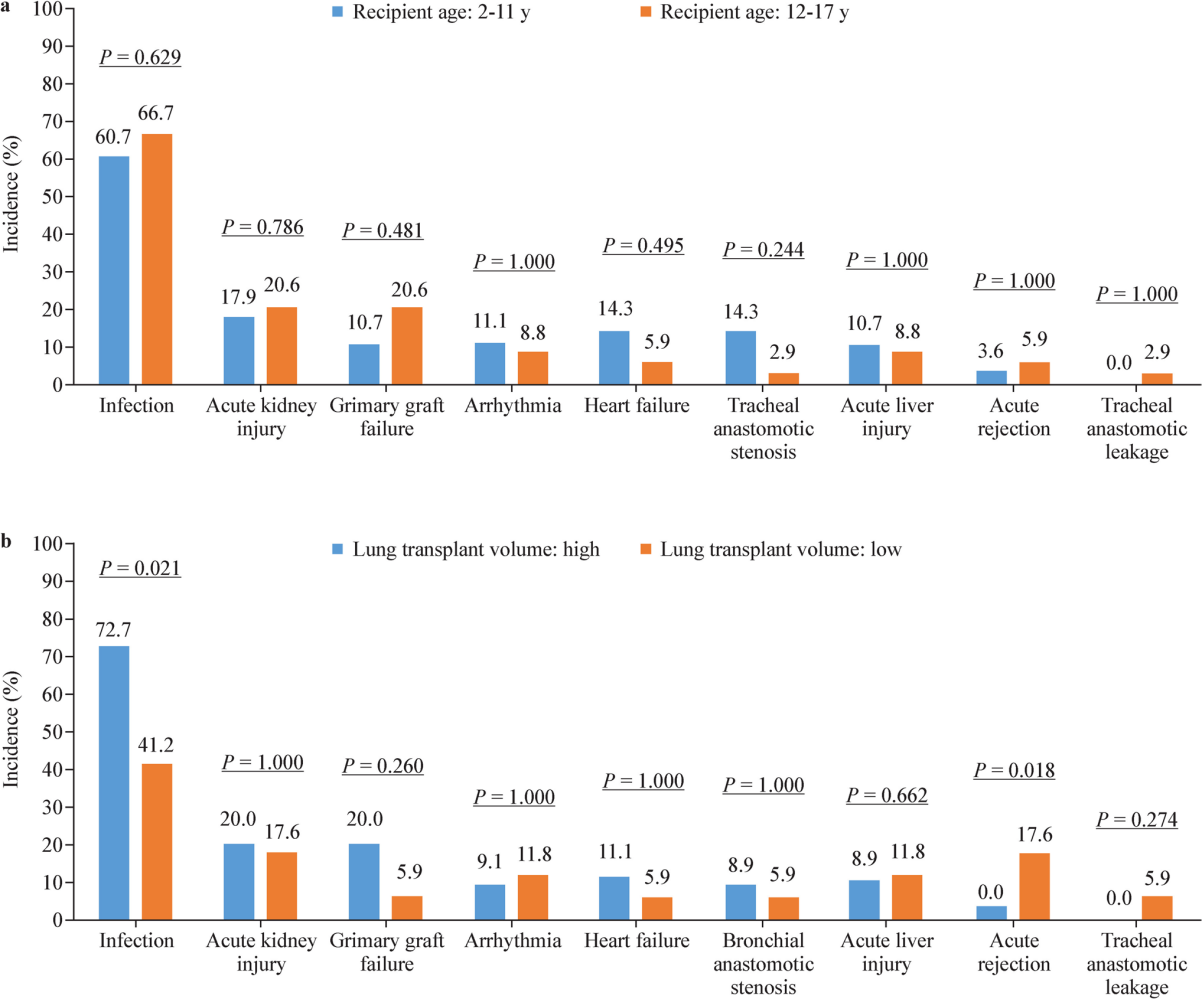
Incidence of postoperative complications in pediatric lung transplant recipients in China from 2019–2023. **a** Comparison by age group; **b** comparison by lung transplant volume

Shown in Fig. 3, the most common complication following pLTX before hospital discharge was infection (63.9%), followed by acute kidney injury (19.4%), primary graft failure (16.1%), arrhythmia (9.8%), heart failure (9.7%), acute liver injury (10.0%), bronchial anastomotic stenosis (8.1%), acute rejection (4.8%), and bronchial anastomotic leakage (1.6%). The incidence of bronchial anastomotic stenosis in recipients aged 2–11 years was greater than that in recipients aged 12–17 years (14.3% vs. 2.9%, *P* = 0.244) (Fig. 3a).

Nine recipients underwent reintubation, which predominantly occurred within the perioperative period, typically within two weeks after the initial extubation. The primary causes for reintubation were respiratory muscle weakness leading to insufficient cough and sputum clearance and inadequate tidal volume for extubation (*n* = 4), primary graft dysfunction (*n* = 2), surgical complications, including tracheal anastomotic leakage and lung atelectasis (*n* = 1), postoperative infection (*n* = 1), and pulmonary edema secondary to renal insufficiency (*n* = 1). The in-hospital mortality among reintubated patients was notably high at 44% (4/9), significantly exceeding the rate observed in patients who did not require reintubation (9.8%, 5/51).

### Outcomes after pediatric lung transplantation

The median follow-up time of the 62 pediatric recipients was 425.5 (IQR = 337.8–1005.8, range: 4–1756) days. The 30-day, 180-day, and one-year survival rates and 95% CIs after surgery were 93.6% (87.6–99.9), 80.6% (71.4–91.1) and 80.6% (71.4–91.1), respectively.

**Fig. 4:**
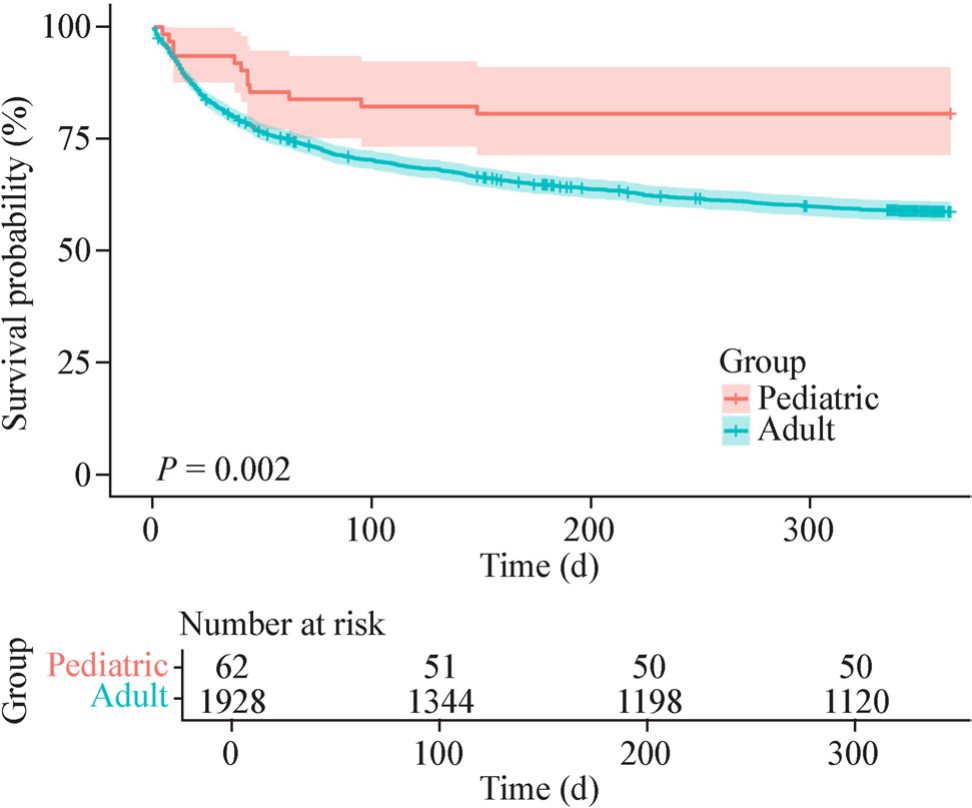
Comparison of one-year postoperative survival rates between pediatric and adult lung transplantation patients

The one-year survival rate of pLTX patients was significantly greater than that of aLTX patients (80.6% vs. 58.7%, *P* = 0.002) (Fig. 4). The in-hospital mortality and one-year mortality rates did not differ by LTX volume, year, recipient sex, recipient age, indications, donor age, cold ischemia time, or ECMO usage during surgery (Table 5, all *P* values > 0.05).

**Table 5:** Comparison of in-hospital mortality and one-year mortality among pediatric lung transplant recipients with different characteristics

Characteristics	Groups	In-hospital mortality		One-year mortality	
		RR (95% CI)	*P*	HR (95% CI)	*P*
Lung transplant volume	Low (*n* = 17)	Reference		Reference	
	High (*n* = 45)	2.39 (0.33–17.18)	0.387	1.46 (0.32–6.65)	0.628
Year of LTX	2019–2021 (*n* = 29)	Reference		Reference	
	2022–2023 (*n* = 33)	1.14 (0.37–3.54)	0.823	1.20 (0.38–3.79)	0.754
Recipients’ sex	Male (*n* = 30)	Reference		Reference	
	Female (*n* = 32)	1.41 (0.44–4.50)	0.566	1.43 (0.45–4.50)	0.544
Recipient age (y)	2–11 (*n* = 28)	Reference		Reference	
	12–17 (*n* = 34)	0.82 (0.26–2.56)	0.737	0.83 (0.27–2.56)	0.742
Indications	Others (*n* = 35)	Reference		Reference	
	Bronchiolitis obliterans (*n* = 27)	0.86 (0.27–2.76)	0.805	0.60 (0.18–1.99)	0.404
Donor age (y)	1–17 (*n* = 39)	Reference		Reference	
	≥ 18 (*n* = 23)	0.19 (0.03–1.39)	0.102	0.31 (0.07–1.40)	0.126
Cold ischemia time (h)	< 8 (*n* = 35)	Reference		Reference	
	≥ 8 (*n* = 27)	1.30 (0.42–4.03)	0.654	1.27 (0.41–3.93)	0.682
ECMO usage	No (*n* = 19)	Reference		Reference	
	Yes (*n* = 43)	3.98 (0.54–29.21)	0.175	5.51 (0.71–42.68)	0.102

*LTX* Lung transplantation, *ECMO* extracorporeal membrane oxygenation, *RR* relative risk, *HR* hazard ratio, *CI* confidence interval

Five pediatric patients underwent lobar LTX. Among these patients, three developed postoperative pulmonary infections, one developed tracheal anastomotic stenosis 95 days after surgery, and one developed renal injury 11 days after surgery. Two patients died postoperatively, one on day 7 and the other on day 148 after surgery, while the remaining three patients survived. None of the patients experienced delayed chest closure.

**Fig. 5:**
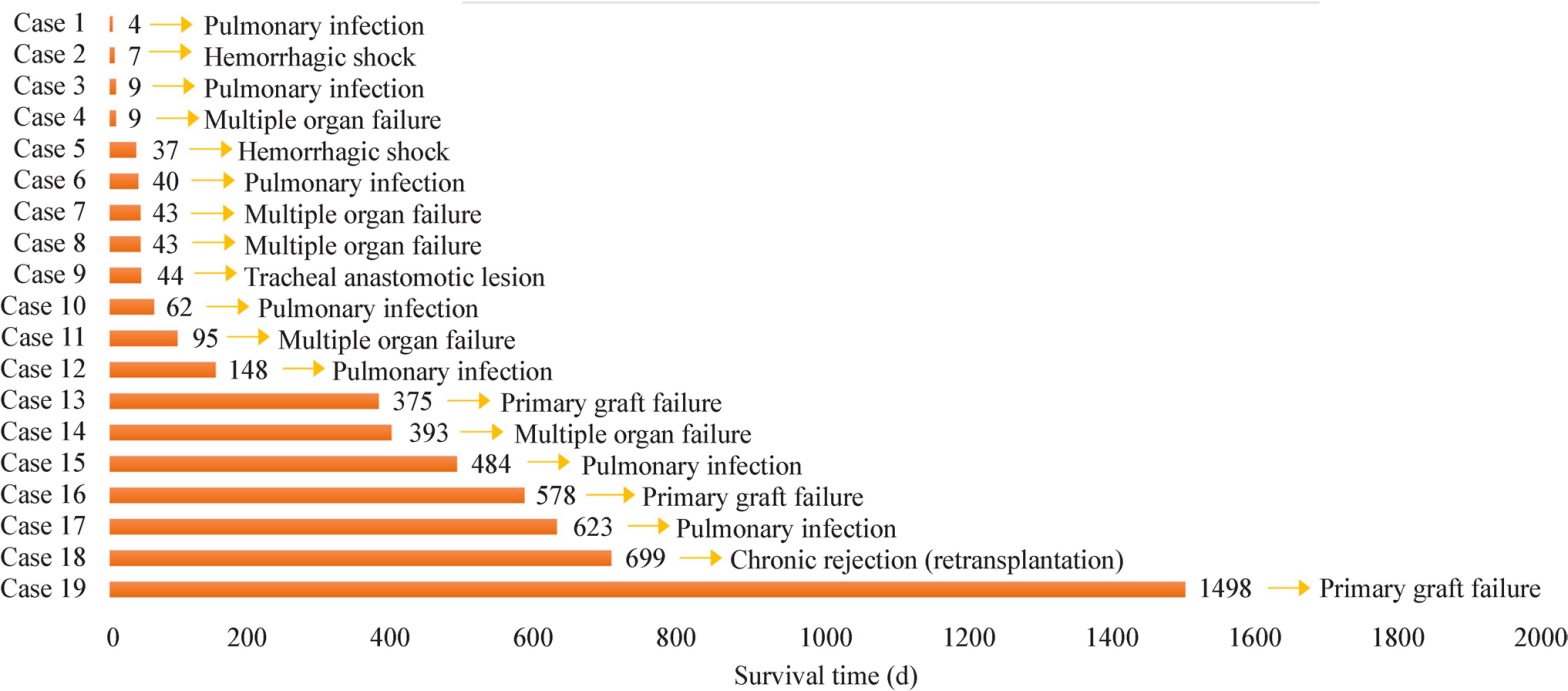
Causes of graft death among 17 pediatric recipients during follow-up

During the follow-up, 18 patients died, and one received lung retransplantation due to chronic rejection, resulting in 19 graft deaths. The follow-up time until death or retransplantation ranged from 4 to 1756 days. As shown in Fig. 5, the most common cause of patient death (graft death) was pulmonary infection (36.8%), followed by multiple organ failure (26.3%), primary graft failure (15.8%), hemorrhagic shock (10.5%), bronchial anastomotic lesions (5.3%), and chronic rejection (5.3%).

A 12-year-old patient who underwent a second LTX was admitted with a primary diagnosis of BOS. The patient underwent the first LTX on January 16, 2021, at the Second Affiliated Hospital of Zhejiang University School of Medicine, with sequential bilateral LTX performed. The postoperative ICU stay was 120 hours. Owing to chronic rejection, the patient underwent retransplantation on December 16, 2022, at the same institution, which was a right single LTX. The postoperative ICU stay was 113 hours. The patient was discharged on January 11, 2023, and remained alive during the most recent follow-up on January 31, 2024.

Among the four cases of early mortality (time from pLTX surgery to death < 30 days), the causes of death included pulmonary infection (*n* = 2), hemorrhagic shock (*n* = 1), and multiple organ failure (*n* = 1). Of the ten in-hospital deaths, five were due to pulmonary infections (50%), three were due to surgical issues (including two cases of hemorrhagic shock and one case of tracheal anastomosis complications) (30%), and two were due to multiple organ failure (20%). Among the seven post-discharge deaths (or graft deaths), four were due to primary graft failure, two were due to multiple organ failure, and one was due to pulmonary infection.

### Comparison of the postoperative characteristics of patients in transplant centers with different transplant volumes

There were no statistically significant differences between high-volume hospitals and low-volume hospitals in terms of duration of hospitalization, ICU length of stay, time between surgery and resumption of diet, duration of ECMO usage after surgery, proportion of reintubation, or duration of reintubation (all *P* > 0.05). However, there was a significantly greater proportion of patients who required ECMO postoperatively in low-volume hospitals than in high-volume hospitals (70.6% vs. 37.8%, *P* = 0.021). With respect to postoperative complications, a greater incidence of infections (72.7% vs. 41.2%, *P* = 0.021) and primary graft failure (20.0% vs. 5.9%, *P* = 0.260) was observed in high-volume hospitals than in low-volume hospitals. However, acute rejection was exclusively observed in low-volume hospitals (0.0% vs. 17.6%, *P* = 0.018) (Fig. 3b). In terms of survival outcomes, no significant associations were observed between transplant volume and in-hospital mortality or one-year mortality (all *P* > 0.05) (Table 6).

**Table 6:** Postoperative characteristics of pediatric lung transplant recipients overall and by lung transplant volume in China, 2019–2023

Characteristics	Total (*N* = 62)	Lung transplant volume		Statistic	*P*
		High (*n* = 45)	Low (*n* = 17)		
Discharged alive, *n* (%)	52 (83.9)	37 (82.2)	15 (88.2)	–	1.000
Hospitalization duration (d), median (IQR)	33.5 (21.0–54.0)	34.0 (23.0–48.0)	30.0 (17.0–66.5)	0.283	0.777
ICU length of stay (d), median (IQR)	6.7 (3.7–13.9)	5.6 (3.6–13.6)	10.0 (5.8–14.5)	0.910	0.363
Time between surgery and resumption of diet (h), median (IQR)	41.0 (24.0–72.0)	40.0 (24.2–70.8)	48.0 (18.0–144.0)	0.445	0.657
ECMO usage after surgery, *n* (%)	29 (46.8)	17 (37.8)	12 (70.6)	5.335	0.021
Duration of ECMO usage after surgery (h), median (IQR)	32.0 (16.0–133.5)	34.0 (13.5–154.5)	32.0 (24.0–84.0)	0.637	0.524
Reintubation, *n* (%)	11 (17.7)	7 (15.6)	4 (23.5)	0.130	0.718
Duration of reintubation (d), median (IQR)	10.5 (3.7–26.0)	10.5 (5.9–24.3)	11.2 (2.3–28.4)	14.000	1.000

*ICU* intensive care unit, *ECMO* extracorporeal membrane oxygenation, *IQR* interquartile range

## Discussion

This study described the trends and outcomes of pLTX in China from 2019–2023. While the annual number of pLTX has remained low, there has been a gradual increase each year. The primary indications for pLTX were BOS, CF, and IPAH. Infection is the most common complication after pLTX [11], and the incidence of bronchial anastomotic stenosis is higher among younger patients than among older children. A higher incidence of infections and primary graft failure were observed in hospitals with high volumes of pLTX, whereas acute rejection was exclusively observed in low-volume hospitals. The one-year survival following LTX in pediatric patients was greater than that in adults, but the long-term outcomes after pLTX require further investigation in long-term follow-up studies.

pLTX, as an invaluable approach for the treatment of children with end-stage lung disease, can effectively improve the survival and quality of life of children [12, 13]. In recent years, with the advancement of LTX techniques and increasing experience, pLTX has been performed in several LTX centers in China, although the number of cases remains relatively low. The annual absolute number of pLTX performed is significantly lower than that of aLTX each year in China, with only approximately 1.8% of the total number of LTX performed in children. However, the annual number of pLTX increased by 144% from 2019–2023, which is greater than the increase in aLTX (105%), suggesting the rapid development of pLTX in China over the past five years.

In China, pLTX is performed predominantly in two transplant centers, and seven of the 13 hospitals reported that only one pLTX was performed between 2019 and 2023, indicating a potential geographic disparity in pLTX accessibility and donor lung availability. For complex procedures such as LTX, patients often prefer to choose hospitals with extensive transplant experience, especially when a referral is not needed. Wuxi People’s Hospital and the Second Affiliated Hospital of Zhejiang University School of Medicine are the largest LTX centers in China. Consequently, most pLTX patients receive LTX at these experienced hospitals, leading to a significant number of pLTX surgeries. Although there are no specialized pLTX surgeons in China, pediatric cardiac or pediatric pulmonary surgeons are generally involved as surgical assistants or consultants during these procedures. As other qualified LTX centers accumulate more experience with pLTX, the current concentration of pLTX procedures is expected to gradually become more distributed across multiple hospitals.

We explored the impact of transplant volume on the postoperative management and prognosis of pLTX. Overall, we did not observe an association between transplant volume and in-hospital mortality or one-year mortality. However, we found that transplant volume was correlated with certain postoperative characteristics and complications in pediatric recipients. Compared with high-volume hospitals, low-volume hospitals had a significantly higher rate of postoperative ECMO usage. These findings suggest that high-volume hospitals achieve better immediate postoperative outcomes for pLTX patients, likely because of their more extensive surgical experience. Additionally, low-volume hospitals tend to adopt more conservative post-transplant maintenance strategies, potentially leading to prolonged ECMO usage. High-volume centers also benefit from well-established immunosuppressive management, which contributes to a lower incidence of acute rejection. Interestingly, we also observed that the incidence of infections and graft failure was greater in high-volume hospitals. This may be because low-volume hospitals often have dedicated specialized teams for pLTX, allowing for more meticulous management and thereby reducing the occurrence of cross-infections and graft failure.

According to the ISHLT, the indications for pLTX depend on age [14]. CF is the primary disease leading to pLTX among patients over 5 years of age, and it is more common in Europe than in North America or other regions [15]. Between 2000 and 2018, CF was the indication for pLTX among 65% of children aged ≥ 11 years and 48% of children aged 6–10 years, whereas in 1- to 5-year-old patients, IPAH was the leading indication for pLTX (27%), and CF accounted for only 3.4%. However, in this study, the most common indication among children aged 2–17 years was BOS (46.0%), followed by CF (12.7%) and IPAH (11.1%). In China, the primary diseases resulting in pLTX are different from those in Europe and America [4], which may be attributable to the difference in the epidemiology of indications. CF is more frequently diagnosed among Caucasian populations but relatively rare in Asian populations [16, 17], with fewer than thirty cases identified in China in recent years [18]. To date, there have been no population-based studies reporting the prevalence of CF in China. The incidence of CG in China is estimated to be 1 in 128,434 to 1 in 64,000 [19, 20], suggesting that CF is a rare disease in China, whereas in European and American populations, the incidence is approximately 1 in 6000 to 1 in 3000 [21, 22]. In 2012, the U.S. Food and Drug Administration approved the first CF transmembrane conductance regulator (CFTR) modulator, the combination therapy ivacaftor/lumacaftor (Orkambi), for treating CF patients with the F508del gene mutation, which has significantly improved survival in CF patients. However, Orkambi has shown suboptimal clinical outcomes for patients with other concurrent mutations, and the F508del mutation is common in Europe and the U.S. but very rare in the Chinese population [23]. The most common genetic mutation in Chinese pediatric CF patients is c.2909G > A, whereas the c.1766 + 5G > T mutation, which is frequently found in Chinese CF patients, is rare among Caucasians [24].

CFTR modulators such as Ivacaftor (Kalydeco), Lumacaftor/Ivacaftor (Orkambi), and Tezacaftor/Ivacaftor (Symdeko) target various CFTR protein defects caused by different gene mutations. However, Kalydeco is primarily used to treat CF patients with the G551D mutation, Orkambi is specifically designed for patients carrying two copies of the F508del mutation, and Symdeko is also primarily applied to F508del mutation carriers. Currently, no targeted therapies for children with CF in China are available on the market. In China, the long-term management of CF in children has focused mainly on airway clearance, anti-inflammatory treatments, and the prevention and eradication of infections. Despite these treatments, some children experience rapid disease progression and ultimately require LTX. Among the eight CF patients who underwent LTX in this study, one died due to postoperative infection, while the remaining patients survived.

In our study, we did not observe a significant difference in the indications for pLTX according to age group, probably because of the small sample size. The percentage of pLTX performed in children aged 12 years and over in our study was approximately 54.8% (34/62). LTX in children of different ages presents distinct challenges in China, particularly with respect to surgical techniques and perioperative management. All pLTX in China are performed by teams that usually perform aLTX. For younger children, the size of anastomoses is different from that of aLTX, which increases the complexity of the procedure. In terms of perioperative management, younger patients require more precise administration of anesthetic agents and fluid management to protect newly transplanted lungs, specifically to avoid overventilation, which could lead to barotrauma. LTX in older children also poses unique challenges. Older pediatric patients may receive adult donor lungs or undergo lung volume reduction surgery, making it more difficult to address anastomotic mismatch. Additionally, the primary indication in older children is BOS due to graft-versus-host disease, which is different from those typically seen in younger patients. Many older pediatric recipients have undergone hematopoietic stem cell transplantation and prolonged chemotherapy, resulting in poor physical function, malnutrition, and some degree of hepatic and renal dysfunction, which complicates overall treatment.

In terms of surgical techniques, there are differences in the choice of sutures for pediatric patients across different age groups, although the differences in anastomotic trimming and vascular anastomosis are minimal. The selection of sutures primarily depends on the child’s size and the size of the anastomosis. Generally, for patients under 12 years of age, 5–0 or 6–0 sutures are used, whereas older children typically require 4–0 or 5–0 sutures. For anastomotic trimming, the general principle is to preserve as much of the recipient bronchus as possible. Typically, only one or two cartilage rings of the donor bronchus are retained at the first bifurcation, but preserving the surrounding bronchial soft tissue to prevent bronchial anastomotic leakage is essential. In experienced LTX centers, vascular anastomosis rarely presents difficulties or complications, with the primary concern being the avoidance of vascular torsion. A vascular mismatch between the donor and recipient is a common issue in pLTX, but it is usually resolved through vascular plasty.

In patients who undergo LTX with concurrent cardiac defect repair, a clamshell incision is used; however, this approach is primarily performed in adult patients. We previously published relevant papers on the concurrent repair of cardiac defects during LTX, particularly in cases involving pulmonary hypertension and isolated congenital heart defects [25]. For pediatric patients with complex congenital heart disease, combined heart–lung transplantation may be considered, although this approach is typically limited to case reports. The choice of incision for the pLTX usually involves a front-lateral incision or bilateral posterior-lateral incisions. In cases of combined heart–lung transplantation, median sternotomy is generally used. In our study, two pediatric patients underwent heart–lung transplantation. The number of heart–lung transplants performed in China is extremely limited, primarily due to the high degree of surgical complexity and the difficulty in donor organ allocation. Heart–lung transplantation is typically reserved for patients with complex congenital heart defects or severe pulmonary hypertension with a right-to-left shunt.

ECMO facilitates oxygen and carbon dioxide exchange through either a V-V or V-A circuit, allowing for a decrease in the intensity of mechanical ventilation, thereby reducing the risk of barotrauma to the lungs and improving gas exchange [26]. Although ECMO is a high-risk invasive procedure associated with various complications, including bleeding, thromboembolism, neurologic injury, infection, and renal failure, it is increasingly utilized as a bridging solution for children on the waitlist for LTX who do not respond adequately to conventional respiratory support [13, 26, 27]. The use of ECMO as a bridge for intra- or postoperative transplantation was not found to negatively impact postoperative survival rates in pediatric patients. In previous studies, survival to hospital discharge and one- and three-year survival rates of pediatric patients who received ECMO as a bridge for LTX were not significantly different from those of patients who received preoperative ECMO in the non-ECMO group [26, 28, 29]. In a cohort of 954 children under 18 years who underwent LTX with 40 patients on ECMO, post-transplant survival was not different between patients who received ECMO at the time of LTX and those who did not [30]. In our study, ECMO support was performed in 6.5%, 69.4%, and 46.8% of pediatric patients at the pre-, intra-, and post-operative stages, respectively. We observed that the use of ECMO was comparable between older and younger pediatric patients before (8.8% vs. 3.6%) and during surgery (76.5% vs. 60.7%) but was much more common among older children than among younger patients (61.8% vs. 28.6%) after the operation, indicating that older children require the maintenance of ECMO support after surgery. This may be because, compared with younger patients, those aged 12–17 years experienced more challenging intraoperative procedures, as indicated by longer operation times and greater volumes of blood loss. In prior research, patients requiring post-transplant ECMO received more transfusions [31]. However, owing to the small sample size in the current study, we were underpowered to study the factors associated with ECMO use or its outcomes among pediatric patients. Further investigations with larger sample sizes are needed to understand the risk factors and impacts of ECMO in pLTX patients.

In recent years, overall survival following pLTX has progressed, resulting in favorable long-term outcomes comparable to those of aLTX, which may be attributed to advances in the pretransplant, peritransplant, and long-term management of recipients [32]. According to data from the ISHLT Thoracic Transplant Registry, the median survival of children after LTX was 5.5 years between 1990 and 2016 [14]. In addition, higher early mortality in pediatric recipients and favorable long-term survival in adults after LTX were reported by the ISHLT [14]. However, in the current study, we observed a higher survival rate within one year following LTX among pediatric patients than among adult patients. The difference in short-term outcomes following pLTX between China and other countries may be due to discrepancies in the indications for pLTX and donor and recipient characteristics. In China, the most common indication for pLTX is BOS due to hematopoietic stem cell transplantation, whereas the main indication for aLTX is interstitial fibrosis. Although the specific postoperative survival rate of LTX for BOS due to hematopoietic stem cell transplantation (HSCT) is limited in China, the postoperative survival rate of LTRs with BOS due to HSCT is approximately 88% at 2 years and 79% at 3 years in other countries [33]. However, the postoperative 1-year survival rate of LTRs with interstitial fibrosis in China is 67%, and the 3-year survival rate is 58%. Therefore, the difference in indications may contribute to the relatively higher survival rates observed for pLTX than for aLTX in China. In addition, according to the principle of lung allocation in China, children under 12 years of age are prioritized on the waiting list according to medical urgency, allowing for quicker access to suitable donor lungs than adults do.

Notably, the survival rates of aLTX patients are still lower in China (81.45%, 70.11%, and 61.16% at 30 days, 1 year, and 3 years, respectively) than those reported by the ISHLT (89% at 3 months, 80% at 1 year, and 65% at 3 years, respectively) [34, 35]. Similar to the results from the ISHLT, we did not observe statistically significant differences in survival by age group [14]. To date, evidence on the impact of donor allograft age on the outcomes of pLTX has been conflicting [36]. Hayes et al*.* reported that adult donor lung allografts did not negatively affect survival in pLTX recipients [29], whereas in a single-center study, a donor age of more than 16 years was associated with poorer survival in pLTX patients [36]. Owing to the small sample size in our study, we were not able to examine the associations between donor age and survival outcomes in pLTX recipients. In addition, owing to the limited follow-up time, we were unable to compare long-term survival between children and aLTX patients. Future studies are needed to understand the long-term outcomes following pLTX in China.

Previous studies have identified several risk factors for anastomotic complications after pLTX, including donor and recipient factors, indications, surgical anastomosis techniques, immunosuppressive therapy, and infections [37, 38]. In our study, the incidence of bronchial anastomotic stenosis after pLTX was greater among younger patients than among older children, which may be attributed to the small diameter of the airway among younger children, which may be a risk factor for the development of stenosis. According to the 2018 ISHLT consensus statement on airway complications in adult and pediatric lung transplantation [39], current treatment strategies for bronchial stenosis typically involve balloon dilation, bronchial stent placement, laser therapy, argon plasma coagulation, and cryotherapy [40–42]. In China, balloon dilation is the first-line treatment for pediatric patients with bronchial stenosis, followed by adjunctive therapies such as cryotherapy and laser treatment to clear the airway. For patients who do not respond well to repeated balloon dilations, stent placement is considered an alternative therapeutic option.

By comparing the causes of early mortality, in-hospital mortality, and post-discharge mortality following pLTX, we found that in-hospital deaths were attributed primarily to infection and surgical factors, whereas postdischarge mortality was attributed predominantly to primary graft failure. This pattern closely mirrors the causes of early mortality in aLTX, suggesting that certain aspects of post-transplant management in pLTX could benefit from the experiences and strategies used in aLTX. Moreover, although not statistically significant, infection was more common among younger recipients following pLTX, further suggesting an increased risk of anastomotic complications among younger children. Given that airway anastomotic complications remain important risk factors for morbidity and mortality, active monitoring and early recognition of such complications are necessary for improving patient outcomes and quality of life following pLTX [11].

There are several limitations in this retrospective study. Only descriptive analyses were feasible because of the relatively small sample size. In particular, multivariable negative binomial or multivariate Cox regression analyses were underpowered because of the small number of events (10 in-hospital deaths and 12 one-year deaths). Thus, studies on long-term survival and the factors influencing mortality in pLTX recipients are needed in the future. However, our study utilized the most comprehensive data currently available from China’s largest registry of pLTX recipients, offering valuable insights into the characteristics and outcomes of pLTX over the past five years, thereby contributing up-to-date and practical experience to the field of pLTX in China.

In conclusion, this study highlights the trends, indications, patient characteristics, and prognoses of pLTX in China from 2019–2023. Although limited in scale, pLTX shows signs of steady growth accompanied by favorable survival outcomes. Further studies examining factors associated with long-term outcomes following pLTX are needed for pre- and post-transplantation medical decision-making to improve quality of life and reduce mortality risks among pediatric recipients. Notably, there is a lack of guidelines for pLTX in China in terms of candidate recipient selection, donor lung allocation, or postoperative care. Given that the field of pLTX in China is still in its early stages, there is a substantial opportunity for development and progress in this critical area of life-saving treatment for children with end-stage lung diseases.

## Supplementary Information

Below is the link to the electronic supplementary material.

Supplementary file 1 (PDF 219 KB)

## Data Availability

The data that support the findings of this study are available from the China Lung Transplantation Registry (CLuTR, https://clutr.cotr.cn/login.jsp) but restrictions apply to the availability of these data, which were used under license for the current study, and so are not publicly available. Data are however available from the authors upon reasonable request and with permission of the Chinese Lung Transplantation Quality Management and Control Center.

## References

[1] Chambers DC, Perch M, Zuckermann A, Cherikh WS, Harhay MO, Hayes D Jr, et al. The International Thoracic Organ Transplant Registry of the International Society for Heart and Lung Transplantation: thirty-eighth adult lung transplantation report—2021; focus on recipient characteristics. J Heart Lung Transplant. 2021;40:1060–72. 34446355 10.1016/j.healun.2021.07.021 PMC10285650

[2] Wu B, Hu C, Chen W, He J, Jiang G, Zhang J, et al. China lung transplantation developing: past, present and future. Ann Transl Med. 2020;8:41. 32154286 10.21037/atm.2019.10.26 PMC7036632

[3] Singh TP, Cherikh WS, Hsich E, Harhay MO, Hayes D Jr, Perch M, et al. The International Thoracic Organ Transplant Registry of the International Society for Heart and Lung Transplantation: twenty-fifth pediatric heart transplantation report—2022; focus on infant heart transplantation. J Heart Lung Transplant. 2022;41:1357–65. 35989143 10.1016/j.healun.2022.07.019 PMC10281815

[4] Yue B, Wu B, Zhang J, Xu H, Wei D, Hu C, et al. Pediatric lung transplantation in the largest lung transplantation center of China: embarking on a long road. Sci Rep. 2020;10:12471. 32719472 10.1038/s41598-020-69340-0 PMC7385630

[5] Medical Emergency Department of National Health Commission of the People’s Republic of China. Notice of the National Health Commission on issuing the “Regulations on the registration and administration of human organ transplantation diagnosis and treatment subjects”. http://www.nhc.gov.cn/ylyjs/pqt/202405/5102d68c35de488986ee889f3aa3d1bc.shtm. Accessed 20 Jan 2025.

[6] Van Raemdonck D, Neyrinck A, Verleden GM, Dupont L, Coosemans W, Decaluwé H, et al. Lung donor selection and management. Proc Am Thorac Soc. 2009;6:28–38. 19131528 10.1513/pats.200808-098GO

[7] Branch of Organ Transplantation of Chinese Medical Association, National Quality Management and Control Center for Lung Transplantation. Guideline on the standard of lung transplantation donors and the acquisition and transshipment in China. Organ Transplantation. 2018;9:325–33 (**in Chinese**).

[8] Branch of Organ Transplantation of Chinese Medical Association, National Quality Management and Control Center for Lung Transplantation. Guideline on the application of extracorporeal membrane oxygenation during the perioperative period of lung transplantation (2019 edition). Organ Transplantation. 2019;10:402–9 (**in Chinese**).

[9] Consensus Writing Group of Pediatric Extracorporeal Membrane Oxygenation, Subspecialty Group of Critical Care Medicine, the Society of Pediatrics, Chinese Medical Association. Experts consensus on the application of extracorporeal membrane oxygenation in critically ill children. Zhonghua Er Ke Za Zhi. 2022;40:183–91 (**in Chinese**). 10.3760/cma.j.cn112140-20211108-00934 35240736

[10] Martin AK, Mercier O, Fritz AV, Gelzinis TA, Hoetzenecker K, Lindstedt S, et al. ISHLT consensus statement on the perioperative use of ECLS in lung transplantation: Part II: Intraoperative considerations. J Heart Lung Transplant. 2024. 10.1016/j.healun.2024.08.027. 39453286 10.1016/j.healun.2024.08.027

[11] Sathe N, Beech P, Croft L, Suphioglu C, Kapat A, Athan E. *Pseudomonas aeruginosa*: infections and novel approaches to treatment “Knowing the enemy” the threat of *Pseudomonas aeruginosa* and exploring novel approaches to treatment. Infect Med (Beijing). 2023;2:178–94. 38073886 10.1016/j.imj.2023.05.003 PMC10699684

[12] Benden C. Pediatric lung transplantation. J Thorac Dis. 2017;9:2675–83. 28932575 10.21037/jtd.2017.07.84 PMC5594176

[13] Benoit TM, Benden C. Pediatric lung transplantation: supply and demand. Curr Opin Organ Transplant. 2019;24:324–8. 31090643 10.1097/MOT.0000000000000630

[14] Rossano JW, Cherikh WS, Chambers DC, Goldfarb S, Hayes D Jr, Khush KK, et al. The international thoracic organ transplant registry of the international society for heart and lung transplantation: twenty-first pediatric heart transplantation report—2018; focus theme: multiorgan Transplantation. J Heart Lung Transplant. 2018;37:1184–95. 30293614 10.1016/j.healun.2018.07.018

[15] Rossano JW, Singh TP, Cherikh WS, Chambers DC, Harhay MO, Hayes D Jr, et al. The international thoracic organ transplant registry of the international society for heart and lung transplantation: twenty-second pediatric heart transplantation report–2019; focus theme: donor and recipient size match. J Heart Lung Transplant. 2019;38:1028–41. 31548029 10.1016/j.healun.2019.08.002 PMC6819143

[16] Colombo C, Littlewood J. The implementation of standards of care in Europe: state of the art. J Cyst Fibros. 2011;10(Suppl 2):S7–15. 21658645 10.1016/S1569-1993(11)60003-9

[17] Singh M, Rebordosa C, Bernholz J, Sharma N. Epidemiology and genetics of cystic fibrosis in asia: in preparation for the next-generation treatments. Respirology. 2015;20:1172–81. 26437683 10.1111/resp.12656

[18] Liu Y, Wang L, Tian X, Xu KF, Xu W, Li X, et al. Characterization of gene mutations and phenotypes of cystic fibrosis in chinese patients. Respirology. 2015;20:312–8. 25580864 10.1111/resp.12452

[19] Ni Q, Chen X, Zhang P, Yang L, Lu Y, Xiao F, et al. Systematic estimation of cystic fibrosis prevalence in Chinese and genetic spectrum comparison to Caucasians. Orphanet J Rare Dis. 2022;17:129. 35313924 10.1186/s13023-022-02279-9 PMC8935702

[20] Guo X, Liu K, Liu Y, Situ Y, Tian X, Xu KF, et al. Clinical and genetic characteristics of cystic fibrosis in CHINESE patients: a systemic review of reported cases. Orphanet J Rare Dis. 2018;13:224. 30558651 10.1186/s13023-018-0968-2 PMC6296146

[21] Southern KW, Munck A, Pollitt R, Travert G, Zanolla L, Dankert-Roelse J, et al. A survey of newborn screening for cystic fibrosis in Europe. J Cyst Fibros. 2007;6:57–65. 16870510 10.1016/j.jcf.2006.05.008

[22] Scotet V, Gutierrez H, Farrell PM. Newborn screening for CF across the Globe—Where is it worthwhile? Int J Neonatal Screen. 2020;6:18. 33073015 10.3390/ijns6010018 PMC7422974

[23] Tian X, Liu Y, Yang J, Wang H, Liu T, Xu W, et al. p.G970D is the most frequent *CFTR* mutation in Chinese patients with cystic fibrosis. Hum Genome Var. 2016;3:15063. 27081564 10.1038/hgv.2015.63 PMC4785583

[24] Chen Q, Shen Y, Zheng J. A review of cystic fibrosis: basic and clinical aspects. Animal Model Exp Med. 2021;4:220–32. 34557648 10.1002/ame2.12180 PMC8446696

[25] Wang Z, Jiao G, Huang J, Chen Y, Chen J. Management of Eisenmenger syndrome by cardiac defect closure combined bilateral lung transplantation with intraoperative venoarterial ECMO support. J Card Surg. 2021;36:1560–2. 33491222 10.1111/jocs.15360

[26] Toprak D, Midyat L, Freiberger D, Boyer D, Fynn-Thompson F, Visner G. Outcomes of mechanical support in a pediatric lung transplant center. Pediatr Pulmonol. 2017;52:360–6. 27787952 10.1002/ppul.23535

[27] Abrams D, Brodie D, Arcasoy SM. Extracorporeal life support in lung transplantation. Clin Chest Med. 2017;38:655–66. 29128016 10.1016/j.ccm.2017.07.006

[28] Sainathan S, Ryan J, Sharma M, Harano T, Morell V, Sanchez P. Outcome of bridge to lung transplantation with extracorporeal membrane oxygenation in pediatric patients 12 years and older. Ann Thorac Surg. 2021;112:1083–8. 33217402 10.1016/j.athoracsur.2020.08.083

[29] Hayes D Jr, McConnell PI, Tobias JD, Whitson BA, Preston TJ, Yates AR, et al. Survival in children on extracorporeal membrane oxygenation at the time of lung transplantation. Pediatr Transplant. 2015;19:87–93. 25425268 10.1111/petr.12400

[30] Koh W, Zang H, Ollberding NJ, Ziady A, Hayes D Jr. Extracorporeal membrane oxygenation bridge to pediatric lung transplantation: modern era analysis. Pediatr Transplant. 2023;27:e14570. 37424517 10.1111/petr.14570 PMC10530187

[31] Boffini M, Simonato E, Ricci D, Scalini F, Marro M, Pidello S, et al. Extracorporeal membrane oxygenation after lung transplantation: risk factors and outcomes analysis. Ann Cardiothorac Surg. 2019;8:54–61. 30854312 10.21037/acs.2018.12.10 PMC6379200

[32] Werner R, Benden C. Pediatric lung transplantation as standard of care. Clin Transplant. 2021;35:e14126. 33098188 10.1111/ctr.14126

[33] Bos S, Beeckmans H, Vanstapel A, Sacreas A, Geudens V, Willems L, et al. Pulmonary graft-versus-host disease and chronic lung allograft dysfunction: two sides of the same coin? Lancet Respir Med. 2022;10:796–810. 35512715 10.1016/S2213-2600(22)00001-7

[34] Hu CX, Chen WH, He JX, Jiang GN, Li XS, Wei D, et al. Lung transplantation in China between 2015 and 2018. Chin Med J (Engl). 2019;132:2783–9. 31856048 10.1097/CM9.0000000000000543 PMC6940083

[35] Yusen RD, Edwards LB, Dipchand AI, Goldfarb SB, Kucheryavaya AY, Levvey BJ, et al. The Registry of the International Society for Heart and Lung Transplantation: thirty-third adult lung and heart–lung transplant report—2016; focus theme: primary diagnostic indications for transplant. J Heart Lung Transplant. 2016;35:1170–84. 27772669 10.1016/j.healun.2016.09.001

[36] Cano JR, Cerezo F, Algar FJ, Alvarez A, Espinosa D, Moreno P, et al. Prognostic factors influencing survival rates in children following lung transplantation. Transplant Proc. 2008;40:3070–2. 19010197 10.1016/j.transproceed.2008.09.024

[37] Crespo MM. Airway complications in lung transplantation. J Thorac Dis. 2021;13:6717–24. 34992847 10.21037/jtd-20-2696 PMC8662498

[38] Delbove A, Senage T, Gazengel P, Tissot A, Lacoste P, Cellerin L, et al. Incidence and risk factors of anastomotic complications after lung transplantation. Ther Adv Respir Dis. 2022;16:17534666221110354. 35894432 10.1177/17534666221110354 PMC9340386

[39] Crespo MM, McCarthy DP, Hopkins PM, Clark SC, Budev M, Bermudez CA, et al. ISHLT consensus statement on adult and pediatric airway complications after lung transplantation: definitions, grading system, and therapeutics. J Heart Lung Transplant. 2018;37:548–63. 29550149 10.1016/j.healun.2018.01.1309

[40] Campisi P, Forte V. Pediatric tracheostomy. Semin Pediatr Surg. 2016;25:191–5. 27301607 10.1053/j.sempedsurg.2016.02.014

[41] Funamura JL, Yuen S, Kawai K, Gergin O, Adil E, Rahbar R, et al. Characterizing mortality in pediatric tracheostomy patients. Laryngoscope. 2017;127:1701–6. 27808411 10.1002/lary.26361

[42] Zeng F, Cai L, Guo L, Lan M, Liang J, Gu P. Pulmonary rehabilitation protocols in urgent lung transplantation patients. World J Emerg Med. 2024;15:47–51. 38188546 10.5847/wjem.j.1920-8642.2024.015 PMC10765079

