# Supplementary material for: Pediatric lung transplantation in China, 2019–2023
Source: World J Pediatr. 2025 Jun 3;21(6):597–612. doi: 10.1007/s12519-025-00916-4 (PMC12245931; doi:10.1007/s12519-025-00916-4)
Supplement: Supplementary file 1 — (PDF 219 KB) [file 12519_2025_916_MOESM1_ESM.pdf]

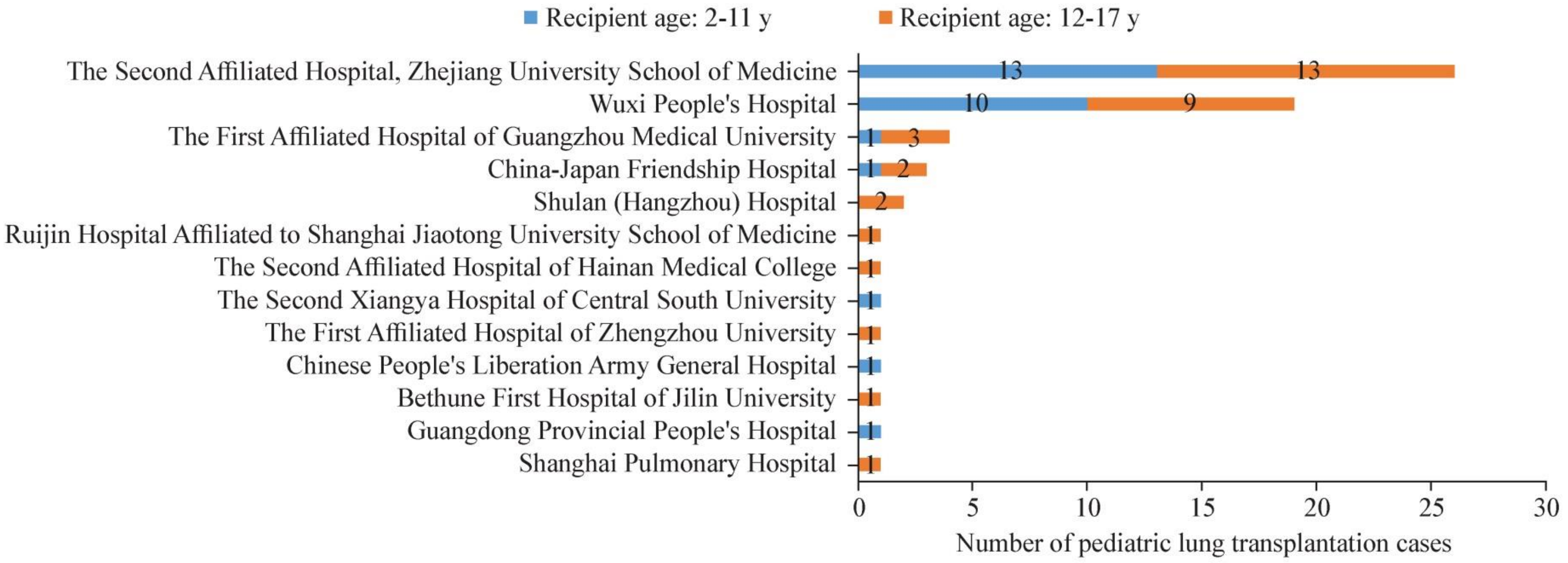

Supplementary Fig. 1 Ranking for hospitals by number of pediatric lung transplants in China from January 2019 to December 2023
